# Catastrophic costs of tuberculosis care: a mixed methods study from Puducherry, India

**DOI:** 10.1080/16549716.2018.1477493

**Published:** 2018-06-14

**Authors:** Thirunavukkarasu Prasanna, Kathiresan Jeyashree, Palanivel Chinnakali, Yogesh Bahurupi, Kavita Vasudevan, Mrinalini Das

**Affiliations:** a Department of Community Medicine and Family Medicine, All India Institute of Medical Sciences, Jodhpur, India; b Department of Community Medicine, Indira Gandhi Medical College and Research Institute, Govt. of Puducherry Institution, Puducherry, India; c Department of Community Medicine, Velammal Medical College Hospital and Research Institute, Madurai, India; d Department of Preventive and Social Medicine, Jawaharlal Institute of Postgraduate Medical Education and Research, Puducherry, India; e Médecins Sans Frontières (MSF)/Doctors Without Borders, New Delhi, India

**Keywords:** health expenditure, coping strategy, patient costs, SORT-IT

## Abstract

**Background**: The average expenditure incurred by patients in low- and middle-income countries towards diagnosis and treatment of TB ranges from $55 to $8198. This out-of-pocket expenditure leads to impoverishment of households. One of the three main targets of the End TB Strategy (2016–2035) is that no TB-affected household suffers catastrophic costs due to TB. Study setting was free care under national tuberculosis program (NTP), Puducherry district, India.

**Objectives**: The objectives of the study were among the newly diagnosed and previously treated tuberculosis (TB) patients, to (a) estimate patient costs during diagnosis and intensive phase of treatment, (b) determine the proportion of households experiencing catastrophic costs, and (c) explore coping strategies.

**Methods**: An explanatory mixed methods design comprising both quantitative cost description and qualitative descriptive component was used. Catastrophic cost was defined as total TB care costs exceeding 20% of annual household income.

**Results**: Of 102 TB patients included, two-thirds (69%) were male, 6% were HIV positive, and 45% reported at least one episode of hospitalization for TB care. The median (IQR) total cost of TB care was US$195 (52.1, 492.9) with a direct cost of US$65.3 (22.3, 156.5) and indirect cost of US$50.2 (0.9, 295.1). Overall, 32.4% of households experienced catastrophic costs due to TB care, significantly higher in patients with HIV coinfection (*p* = 0.009) and hospitalization (*p* = 0.009). Pledging jewels and borrowing money were major coping strategies. Cash assistance was the expected remedy from the patient perspective.

**Conclusion**: Despite free TB care under NTP, more than a third incurred catastrophic costs towards TB care.

## Background

In 2015, tuberculosis (TB) affected an estimated 10.4 million individuals globally, including an estimated 2.8 million cases in India, a high-TB-burden country [1]. Since the burden of TB disproportionately affects the poor, one of the three main targets of the End TB Strategy (2016–2035) is that no TB-affected household suffers catastrophic costs due to TB [2]. This target is a key marker of financial risk protection, progress towards universal health coverage and also an indicator of social protection for TB-affected households.

Evidence suggests that the average expenditure incurred by patients in low- and middle-income countries towards diagnosis and treatment of TB ranges from $55 to $8198 [3]. In a country like India, where government spending on health is low and with a larger presence of private players, patients may be forced to spend out-of-pocket (OOP) to meet the expenditure on health [4]. OOP is the direct payment made by individuals at the time of service use for health services and nonmedical payments (such as transportation, accommodation, food charges), excluding prepayment for health services in the form of taxes or specific insurance premiums [5]. The share of OOP in total health expenditure is a proxy measure of the degree to which people lack financial protection and, when it exceeds 40%, is termed as catastrophic expenditure [5]. Indian Standards for Social Inclusion in TB Care 2014 (Standards 23 and 25) is set to eliminate OOP expenditure through free or affordable and accessible diagnosis and/or treatment and mitigate it through social welfare support respectively [6].

However, in view of the End TB Strategy, it is now recommended to measure catastrophic costs, incurred when the total costs exceed 20% of the annual household income [2]. It is wider than catastrophic expenditure and includes indirect costs such as income loss related to time lost from work or loss of employment in addition to direct costs. Patients and families resort to some coping strategies to balance the costs incurred towards healthcare [3]. The magnitude of the burden posed by the costs and the coping strategies adopted by the families determines the level of impoverishment.

Programmatic data on costs incurred by patients towards TB care and its impact including catastrophic costs are not routinely collected, and limited literature is available from India on the same. Hence in this study, we intended to assess patient costs during diagnosis and treatment of TB patients and explore the perspectives of the patients towards costs for TB care. This will help us devise strategies targeting patient, provider, and community level factors to minimize costs and its impoverishing catastrophic impact. Specific objectives were, among newly diagnosed and previously treated TB patients enrolled under the national TB program in Puducherry district, India between 1 December 2016 and 31 January 2017, (a) to estimate the patients’ costs (direct and indirect) incurred during diagnosis and intensive phase of treatment, (b) to assess the number (proportion) of households experiencing catastrophic costs due to TB, and (c) to explore the patient perspectives regarding costs for TB care and their coping strategies.

## Methods

### Study design

A mixed methods (explanatory) design with cross-sectional quantitative and in-depth qualitative interviews was used. While the quantitative results will estimate the magnitude and pattern of costs, the qualitative results will give us an insight into perspectives of the patients toward costs for TB care and understand the choices they made along their pathway to care.

### General setting

The Puducherry district in Union Territory (UT) of Puducherry, a coastal area in South India, has a population of about 0.95 million, of whom 85% are literate, and 70% reside in urban areas [7]. The annual per capita income is about 172,000 Indian rupees (INR), or approximately US$2600. In 2015–2016, the public per capita expenditure on health in Puducherry was INR 3086 (US$46) compared to national estimate of INR 1042 (US$15.5) in 2013–2014 [7]. As per national data, 3.8% and 3.9% of the total health expenses are covered by government and private health insurance schemes respectively [8].

### TB care in study setting

In 2016, 1415 TB patients were registered for treatment under Revised National Tuberculosis Control Program in UT of Puducherry. Following diagnosis by sputum microscopy, intermittent, short-course chemotherapy (thrice-weekly during intensive phase and once weekly during continuation phase) is provided under direct supervision (thrice-weekly during intensive phase and once weekly during continuation phase) at the nearest peripheral health institution. New cases (Category I) receive DOTS for a duration of 6 months and retreatment cases (Category II) for 8 months. These TB diagnosis and treatment services are offered free of charge through the existing public health care delivery system. There are no TB specific insurance schemes. The Government of Puducherry provides a monthly financial assistance of US$7.5 to elderly/destitute pensioners suffering from TB to meet the medical expenses in addition to the old age pension. TB patients living below poverty line and belonging to socially deprived group (Scheduled Castes) are offered US$30. Besides the public healthcare system, patients can also avail TB care from the private sector, the choice resting with the patient.

### Study participants

All newly diagnosed (Category I) and previously treated (Category II) TB patients of all ages registered for treatment under national TB program in Puducherry district between 1 December 2016 and 31 January 2017 were included. MDR TB patients were not included.

### Sample size and sampling technique

Assuming the mean (SD) of total cost incurred by patients towards TB diagnosis and treatment as INR 3000 (100) [9], a minimum of 104 individuals were required for the study. All eligible participants were approached serially for the study and included until the desired sample size was reached. For qualitative component, perspectives about the costs were captured from five purposively chosen participants with differing levels of costs incurred.

### Data variables and data-collection methods

For quantitative component, study participants were contacted at the end of the intensive phase in person at their home or at health facility as preferred by them. An adaptation of the ‘Tool to Estimate TB Patient’s Costs’ developed by the Poverty Sub-Working group of the StopTB Partnership was used [10]. It was administered after obtaining written informed consent. In the case of pediatric patients (less than 18 years of age), the parents were interviewed. The sociodemographic data and clinical characteristics were extracted from each patient’s TB treatment card and from the TB treatment register. Costs incurred during pretreatment phase (before diagnosis and during diagnosis) and during the intensive phase of treatment were captured.

The direct costs included all OOP costs linked to seeking diagnosis and treatment for TB incurred by patients including medical expenses, fees, hospitalization, transport, accommodation, and food expenditure as well as costs due to comorbidity and food supplements. Indirect costs were cost of foregone income due to inability or reduced ability to work because of the TB illness and loss of time due to visits to health facilities, time spent traveling to and at health facilities, lost productivity, loss of job, lost income, and lost savings. Catastrophic health costs was defined as the total cost of TB care exceeding 20% of the annual income of the household.^2^ The costs were calculated in terms of INR and converted to US dollars (exchange rate at the time of study: US$1 = INR 67.195) [11].

For the qualitative component, in-depth face-to-face interviews were conducted until saturation to obtain patients’ perspectives regarding costs on diagnosis and treatment of TB and coping strategies adopted. Five purposively selected patients were interviewed by a male community physician trained in qualitative methods and from the same linguistic and cultural background as the patients after written informed consent using an interview guide at their respective homes. Audio recording of the interviews was done with the patient’s consent. The recordings were transcribed in English on to paper within 24 hours of the interview.

### Data analysis

The data were single entered and analyzed using EpiData softwares (EpiData Association, Odense, Denmark). Categorical variables such as sociodemographic, clinical, and treatment characteristics were summarized using frequency and proportions. We reported median costs with interquartile range, as the data did not follow normality. Total median (IQR), direct, and indirect costs were calculated per patient and were compared across categories of age, gender, residence, category of TB using the Kruskal–Wallis test. A *p* value of less than 0.05 was considered as statistically significant. A manual content analysis of the transcripts was carried out to look for emerging themes. Two other investigators reviewed the analysis.

### Ethics

The study was approved by the Institutional Ethics Committee of the Indira Gandhi Medical College and Research Institute, Puducherry, India, and the Ethics Advisory Group of the International Union Against Tuberculosis and Lung Disease, Paris, France. Written informed consent was obtained from all participants of the study.

## Results

### Patient profile

A total of 116 patients were approached, and 104 (89.6%) participated. Two interviews were incomplete and hence were excluded. Of 102 patients included for analysis, 46 (45%) were aged between 14 and 44 years, and 70 (69%) were male. A clinical and sociodemographic profile of the participants is presented in Table 1. Among those with complete data in the records, 6 (6.9%) had an HIV coinfection, and 28 (29.8%) had diabetes mellitus. Of the total, 46 (45%) of the patients reported at least one episode of hospitalization for TB care. The median annual income of patients was US$1080, and that of the household was US$1876. The index patient was the prime income earner in 34 (33%) households. Only 5 (4.9%) had any health insurance.

**Table 1.** Sociodemographic and clinical characteristics of patients enrolled for study among those registered for care under NTP, Puducherry, India, December 2016 to January 2017.

Characteristics	No. of patients (%)
**Total**	102 (100)
**Age (years)**	
14–44	46 (45.1)
45–60	32 (31.4)
>60	24 (23.5)
**Gender**	
Male	70 (68.6)
Female	32 (31.4)
**Residence**	
Rural	46 (45.1)
Urban	56 (54.9)
**Education**	
No schooling	23 (22.5)
Primary schooling	26 (25.5)
Secondary schooling	32 (31.4)
Higher secondary	6 (5.9)
College	15 (14.7)
**TB treatment category**	
New	82 (80.4)
Previously treated	20 (19.6)
**Type of TB**	
Pulmonary	80 (78.4)
Extrapulmonary	22 (21.6)
**Comorbidity**	
HIV (*n* = 87)	6 (6.9)
Diabetes (*n* = 94)	28 (29.8)
**Hospitalization**	46 (45.1)
**Type of health facility visited first**	
Government	58 (56.9)
Private^a^	44 (43.1)

^a^Includes one each of Traditional Medicine Provider and Pharmacist.

### Patients’ costs of TB care

The median (IQR) total cost of TB care (pretreatment and treatment during IP) was US$196 (52.1, 492.9). The direct cost accounted for 33% of the total cost. Direct costs were incurred by 100 (98%) patients. Details of direct, indirect and total costs are presented in Table 2.

**Table 2.** Total, direct and indirect costs (US$) incurred for TB diagnosis and intensive phase treatment by patients registered for care under NTP, Puducherry, India, December 2016 to January 2017.

	For study population^a^	For those who incurred the cost	
Costs	Median, IQR	*n*	Median, IQR
**Total**	**195.8 (52.1, 492.9)**	**101**	**199.4 (55.6, 507.7)**
**Direct^b^**	**65.3 (22.3, 156.5)**	**100**	**68.4 (24.3, 156.8)**
Consultation	0 (0, 2.9)	45	3 (1.5, 7.4)
Test + investigation	0.0 (0.0, 8.4)	38	11.2 (7.4, 20.1)
Drugs	0.0 (0.0, 8.9)	45	10.4 (6.7, 29.8)
Travel	5.4 (0.9, 19.4)	93	6.6 (2.1, 20.1)
Food	0.0 (0.0, 6.1)	49	6.6 (1.5, 19.4)
Accommodation	0.0 (0.0, 0.0)	3	89.3 (14.9, 148.8)
Guardian	0.0 (0.0, 1.5)	40	1.7 (1, 11)
Hospitalization	0.0 (0.0, 33.7)	37^c^	49.9 (17.53, 91.9)
Food supplements and others	14.9 (0.0, 48.4)	74	17.9 (11.9, 59.5)
Comorbidity	0.0 (0.0, 0.0)	22	14.1 (6.8, 23.6)
**Indirect**	**50.2 (0.9, 295.1)**	**83**	**133.3 (8.7, 477.8)**
During treatment	0.0 (0.0, 2.3)	44	2.4 (1.2, 5.3)
Hospitalization	0.0 (0.0, 7.3)	26	54.2 (22.3, 105.3)
Guardian	0.0 (0.0, 0.0)	22	0.0 (0.1, 0.4)
Loss due to income change	0.0 (0.0, 44.7)	36	67 (44.7, 107.3)
Loss of productivity	0.0 (0.0, 0.0)	14	2 (0.9, 5.6)
Loss of job	0.0 (0.0, 238.3)	40	334.9 (180.4, 692.7)
Loss of savings^d^	0.0 (0.0, 0.0)	21	8.9 (3.4, 29)

US$ = USA dollars; IQR = interquartile range.

^a^ *n* = 102, median costs of all study participants irrespective of whether they incurred that specific subcomponent cost.

^b^These costs were incurred by the patient in addition to those free services provided by the government for diagnosis and treatment of TB.

^c^Nine patients incurred no costs during hospitalization.

^d^Loss due to interest paid to loan and loss of value of property sold.

Private providers were sought first for diagnosis and pretreatment care by 44 (43%) patients. The median cost incurred by patients who consulted a private provider first was US$196 as against US$221 incurred by those who visited a public facility first.

Among the 83 (82%) who incurred any indirect cost, loss of job (US$334.9), loss due to income change (US$67), and hospitalization (US$54) were the main contributors. At the end of the intensive phase of treatment, 65 (64%) reported being unemployed, and 40 (61.5%) reported TB as a reason for unemployment.

The details of direct, indirect and total costs stratified by clinical and sociodemographic characteristics are presented in Table 3. Direct costs were significantly higher for those patients who were hospitalized (*p* < 0.001) and patients who approached private health facility first (*p* = 0.01). Patients aged 45–60 years, males, HIV-positive patients, and those who were hospitalized had significantly higher indirect costs.

**Table 3.** Overall patient costs (US$) during TB diagnosis and intensive phase treatment according to demographic and select clinical characteristics of patients registered for care under NTP, Puducherry, India, December 2016 to January 2017.

Patient characteristics	*n*	Total patient cost median (IQR)	Direct	Indirect
**Total**		**195.8 (52.1, 492.9)**	**65.3 (22.3, 156.5)**	**50.2 (0.9, 295.1)**
**Age (years)**				
15–44	46	172.4 (48.2, 413.5)	63.6 (29, 157.4)	44.1 (1.2, 200.5)*
45–60	32	338.4 (97.2, 719.1)	69.7 (16.7, 153.3)	228.8 (8.7, 618.7)
>60	24	128.7 (46.6, 353.4)	71.2 (16.9, 179.8)	1.4 (0.0, 207.9)
**Gender**				
Male	70	221.3 (56, 618)	61.5 (18.6, 145.3)	108.3 (2.7, 453.3)*
Female	32	175.4 (49.6, 331.1)	71.5 (45.7, 179.8)	8.9 (0.0, 185.2)
**Residence**				
Rural	46	238.1 (64, 545.1)	59.1 (21.8, 110.1)	113.3 (0.7, 488.5)
Urban	56	190.3 (48.5, 359.9)	76.7 (22.3, 159.1)	27.1 (1, 231.8)
**TB treatment category**				
New	82	195.7 (48.2, 496.8)	66 (21.6, 156.9)	50.2 (0.9, 295.1)
Previously treated	20	198.3 (112, 492.8)	81.1 (31.1, 154.8)	56.1 (0.4, 449.3)
**Type of TB**				
Pulmonary	80	185.3 (48.7, 522.5)	61.5 (20.3, 134.7)	50.4 (1.1, 326.3)
Extrapulmonary	22	243.7 (59.8, 470.9)	100 (28.6, 216.4)	50.2 (0.0, 291.2)
**HIV status**				
HIV positive	6	1112 (407.7, 2282.9)	197.6 (22.3, 718.1)	455.8 (85.6, 150.5)*
HIV negative	81	180.3 (48.4, 437.3)	61.9 (21.1, 116.5)	45.6 (0.8, 282.7)
**Diabetes status**				
Diabetic	28	222.1 (88.5, 513.8)	101.3 (30.8, 184.1)	96.6 (0.9, 370.2)
Nondiabetic	66	186.2 (48.2, 425.3)	62 (19.8, 109.9)	45.4 (0.7, 245.6)
**Hospitalization**				
Yes	46	283.8 (162.4, 887.4)	105.3 (60.1, 216.4)*	137.2 (24, 554.1)*
No	56	97.6 (29.5, 349.4)	45.8 (15.7, 95)	2.89 (0, 216.1)
**Type of health facility first visited^a^**				
Government	58	221.1 (49.1, 492.9)	56 (14.4, 108.9)*	75.7 (2.2, 397)
Private	42	195.8 (63.5, 622.5)	76.4 (46, 218.7)	16.1 (0.0, 236.3)

^a^ *n* = data not available for two records.

Kruskal–Wallis was performed.

*p < 0.05.

TB illness affecting their social and/or private life was reported by 47 (46%) patients.

### Catastrophic costs

In 6 (5.8%) households, total TB costs exceeded the annual household income. The proportion of catastrophic costs calculated using different definitions are shown in Table 4. Taking total TB costs exceeding 20% of the annual household income, 33 (32.4%) households experienced catastrophic costs. The proportion of households experiencing catastrophic costs, stratified by clinical and sociodemographic characteristics, is presented in Table 5. Patients with HIV coinfection and hospitalization were more likely to incur catastrophic costs (*p* < 0.05).

**Table 4.** Proportions of households that incurred catastrophic costs for TB diagnosis and intensive phase treatment of patients registered for care under NTP, Puducherry, India, December 2016 to January 2017, based on different common definitions.

Numerator	Denominator	Cut-off (%)	Percentage of households
Total direct cost	Total annual nonfood expenses	40	16.7
Total direct cost	Total annual household income	10	20.6
Total direct cost	Total annual household income	20	7.8
Total cost	Total annual nonfood expenses	40	38.2
Total cost	Total annual household income	10	49
Total cost	Total annual household income	20	32.4

**Table 5.** Households experiencing catastrophic costs^a^ for TB diagnosis and intensive phase treatment according to demographic and select clinical characteristics of patients registered for care under NTP, Puducherry, India, December 2016 to January 2017.

Patient characteristics	*n*	No. (%) who experienced catastrophic costs	*p*
**Total**	**102**	**33 (32.4)**	
**Age (years)**			
14–44	46	15 (32.6)	0.02
45–60	32	15 (46.9)	
>60	24	3 (32.4)	
**Gender**			
Male	70	25 (35.7)	0.2
Female	32	8 (25)	
**Residence**			
Rural	46	16 (34.8)	0.6
Urban	56	17 (30.4)	
**TB treatment category**			
New	82	25 (30.5)	0.4
Previously treated	20	8 (40)	
**Type of TB**			
Pulmonary	80	26 (32.5)	0.95
Extrapulmonary	22	7 (31.8)	
**HIV status**			
HIV positive	6	5 (83.3)	0.009
HIV negative	81	25 (30.9)	
**Diabetes status**			
Diabetic	28	11 (39.3)	0.31
Nondiabetic	66	19 (28.8)	
**Hospitalization**			
Yes	46	21 (45.7)	0.009
No	56	12 (21.4)	

^a^When total costs exceeded 20% of annual household income.

### Patient perspectives about cost of TB care

**Figure 1.**
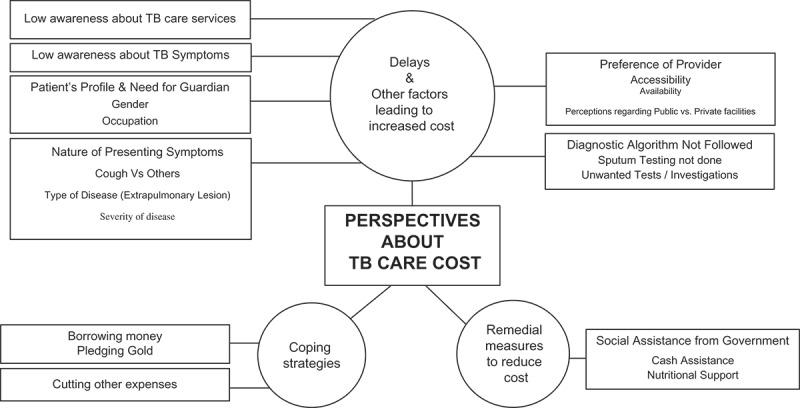
Patient perspectives on TB care cost.

Patient perspectives’ on TB care costs, coping strategies adopted, and remedial measures expected are summarized in Figure 1.

#### Delay in diagnosis and other costs

Patients reported a delay in diagnosis, and thus they had spent more on consultation fee, investigations, treatment, travel, and other related indirect costs.

Initially I consulted with so many private practitioners, but unfortunately all of them failed to detect the presence of TB. Instead I was prescribed tablets which never gave relief for more than a night. (47-year-old female, medium costs)

Patients seemed to understand that TB care is free of cost in public facilities and expressed regret on not being aware at an earlier stage that they were suffering from TB.

If I had known that I have TB, I would have gone to government hospital first as the service is good here. (47-year-old female, medium costs)

I got treatment from Govt. No expenses. (62 year old male, least costs)

Though patients were aware of the availability of TB care facilities in the public hospitals, they had not availed care at first instance there. The primary reasons reported were distance, waiting time, and lack of infrastructure, but none reported cost as a factor.

People are not available when we visit for care. They say they have gone to field or another center. (37-year-old male, medium costs)

It would have been delayed if we had gone to government hospital. (68-year-old female, high costs in the private healthcare)

Earlier it used to be unhygienic and infectious zone but now government hospitals appear clean and hygienic (18-year-old male, high costs in private healthcare)

Patients required a caretaker during the TB illness in the initial phase, which contributed to guardian cost.

I needed one person to go along with me to collect tablets. First two months it was like that. (37-year-old male, medium costs)

### Coping strategies

To cope with the costs for TB care, 38% of TB patients reported borrowing money, and another 8% sold household property. Patients also resorted to pledging jewels or dissolving savings towards meeting costs.

Initially I pledged jewels. Over and above that only, I asked loan from relatives and friends. (37-year-old male, pulmonary TB)

### Remedial measures

The need for government intervention to ease the burden of TB on them was expressed by 78 (76%) patients, with 61 (78%) and 13 (16%) stating a preference in the form of cash assistance and transport/food vouchers respectively. Only 4 (5%) asked for improvement in services in the public hospitals.

## Discussion

This mixed methods study assessed the profile and catastrophic impact of TB care costs on households of TB patients enrolled for care in national TB program. The median total cost incurred towards diagnosis and treatment was US$196; indirect costs accounted for more than 50% of the total cost. Most of the costs were incurred between onset of first symptom and treatment initiation. In total, 32% of households experienced catastrophic costs due to TB care, and it was significantly higher in the presence of HIV coinfection and among those who were hospitalized.

### TB care costs

Despite diagnosis and treatment being available free of cost under the national TB program, the total cost of TB care was high. Zhou *et al*. have reported a total median direct cost of US$637 in rural China despite free TB care being offered [12]. The total median direct cost was estimated at US$41.1 among a tribal population in India by Muniyandi *et al*. [13]. Our estimate was much higher than this Indian study, which was carried out in a setting where active TB case finding strategy was employed, and 86% patients had not incurred any direct cost [13]. Further, 43% of our study population had sought private sector care first as opposed to just 9% in their study. Reasons for higher proportions of participants seeking private sector care are discussed later. Our estimate of direct cost is closer to the pooled estimate of US$61 considered as the direct cost of base care for drug-sensitive TB Care by Verguet *et al*. [14]. Another study from Chennai, India reported a mean total cost of US$48 [9]. Mean total costs for TB care in South Africa and Nigeria were US$207 and $157 respectively [15,16].

Elderly patients had incurred much lower total costs than their younger counterparts due to their negligible indirect costs (median US$1.4). Younger age group (15–60 years) and males understandably incurred maximum indirect TB costs due to lost wages and reduced productivity. Some participants had indirect cost either due to stopped work or due to lost productivity, and our study tool captured both (Table 2). Patients from rural areas experienced 1.2 times higher costs than those from urban areas, due to their disproportionately high indirect costs. Besides distance from healthcare facilities and poor connectivity, rural labor is not irreplaceable, and paid leave is not available, thus making them incur heavy indirect costs due to loss of wages and less productivity. Rural people have also been known to access treatment ‘outside DOTS/NTP’ more often than their urban counterparts [17].

Patients with EPTB experienced higher direct costs than those with pulmonary TB. Qualitative findings support this by pointing to atypical presenting symptoms and thus delay in diagnosis. Patients were unaware and not informed by providers about the possibility of TB as a diagnosis for their symptoms. As previously reported [17], there was a preference of private providers. In India, there is large prevalence of private providers, and the healthcare costs in private care are higher than in the public sector [17,18]. Qualitative interviews revealed that a lack of awareness of TB care service availability in the public sector combined with a lack of awareness about TB symptoms, in the underlying milieu of general preference for private facility, the factors including timings of service availability, perception of quality, etc., could have led to this phenomenon of a higher proportion of participants seeking private-sector care. Further, nonadherence to the TB diagnostic algorithm is common among private practitioners. Unnecessary investigations and symptomatic treatment could have increased the direct costs on TB care [19]. This possibly explains the higher direct costs for those who visited private care first. The median total cost was higher for those who sought government care first. However, mean total costs were lower for who sought government care first.

### Catastrophic costs for TB care

The definition of catastrophic costs is debatable [20]. The first level of uncertainty is choosing which cost to include for estimating catastrophic cost, i.e. the direct cost or the total cost. The second is in defining the denominator from which the total TB cost is met – total income or capacity to pay, i.e. nonfood expenditure after excluding expenditure on basic needs. The third level lies in setting the threshold, beyond which the cost would be termed catastrophic. For our study purposes, we followed the WHO End TB Strategy definition, total cost exceeding 20% of annual household income [2,21]. We have also calculated estimates based on different cut-offs used in other literature to enable comparison.

Incidence of catastrophic costs in China was 66.8% using the 10% of household income measure [12]. Using 40% of nonfood expenditure, it was 54.7% in China and 44% in Nigeria households [12,16]. Both these studies estimated direct costs only without accounting for the indirect costs. Using a 10% of annual household income cutoff as threshold, 71.8% of the patients in Benin, West Africa experienced the catastrophic impact of TB [22]. With total costs ≥20% of household annual income, 39% Peruvian households incurred catastrophic costs [14]. They reported that only this threshold was most strongly associated with adverse TB outcome as against 10% or 15%. We used this threshold and had a comparable yet lower estimate of proportion of those experiencing catastrophic costs, as our study has not included continuation phase of treatment.

HIV coinfection and hospitalization were significantly associated with catastrophic TB costs as reported previously [12,16,23]. Hospitalization increases cost due to direct and indirect patient, and guardian costs involved. In our study, with 45% of the patients having been hospitalized at some point during diagnosis and treatment, this is a concern, as hospitalization for TB is not free under the NTP. A systematic review of patient costs in sub-Saharan Africa identified hospitalization and seeking private sector, among others, as important drivers of catastrophic costs [24]. Unnecessary hospitalizations, not an uncommon practice in the private sector, should not be ignored. Interventions to addressing varying qualities of care at multiple care providers towards access to high-quality care could reduce a good portion of catastrophic cost [14].

### Strengths and limitations

One of the strengths is that we used a validated tool for capturing information on the costs. Second, our quantitative and qualitative findings agreed with each other, thus validating our results. Third, we used different methods for calculating catastrophic costs to derive estimates that can be compared with other studies. There are few limitations. We have studied the costs incurred only during the intensive phase of treatment due to resource limitations. The authors had contacted patients at the end of the intensive phase to enable a better recall of costs that the patient had spent pretreatment and during treatment. We have excluded MDR TB patients from our study due to small numbers who were enrolled during the study period. The magnitude and pattern of costs for MDR TB patients may not be comparable with drug-sensitive TB patients and thus may skew the estimates [14].

Under-reporting of income and over-reporting of expenditure and costs are a common phenomenon. This could have led to overestimating the proportion of households experiencing catastrophic health costs. The objective verification of hospital and pharmacy bills, travel tickets, laboratory bills, etc. would have improved accuracy. However, due to the nonuniform availability of these documents with the patients, the authors refrained from using the same to avoid any bias of differential calculation of costs among subsets of the study population.

Our study participants included only those who were enrolled with the NTP for treatment. There might have been a section of undiagnosed TB patients, those enrolled with the private sector, those on non-DOTS regimens, and those who deferred using services due to prohibitive cost, thus limiting the representativeness of the estimates to the entire population of patients with TB. Differential recall by patients who had longer and complicated pathways to definitive TB treatment could not be ruled out.

### Recommendations

To achieve the ‘END TB strategy’ goal of zero catastrophic costs, there should be an increase in the coverage of community-based health insurance programs. Direct cash-transfer schemes to all TB patients or to those with HIV/TB may help allay the burden. Monitoring of adherence to diagnostic algorithms and hospitalization guidelines for TB diagnosis and treatment, especially among private care providers, would help cut unnecessary delay and costs during diagnosis and treatment. Besides financial barriers, there are knowledge, beliefs, and perception-related barriers that can be fixed by targeting the accessibility, availability, and quality of the primary health care for TB, leading to a further reduction in the cost and the catastrophic impact on the TB-affected household.

## Conclusions

Despite free diagnostic and treatment services offered under the national TB program, the patients incurred considerable costs towards TB care. About a third of TB patients experienced catastrophic costs. HIV coinfection and hospitalization increased the risk of experiencing catastrophic costs due to TB. A comprehensive coverage by social protection mechanisms, preferably as cash assistance, was viewed by the patients as the important remedial measure to be instated.

## Paper context

End TB strategy targets that no patients with TB or their households should face catastrophic costs due to TB. Our study calculated the median total cost of TB diagnosis and treatment in Puducherry, India to be US$196. Despite free TB diagnosis and care services offered under the NTP, more than a third of households of patients with TB incurred catastrophic costs, more likely with HIV coinfection and hospitalization. Cash assistance and regulation of private sector TB care are recommended.
